# Congenital toxoplasmosis: an observational retrospective study in the Eastern Sicily

**DOI:** 10.3389/fped.2025.1597001

**Published:** 2025-06-27

**Authors:** Maria Teresa Garozzo, Rosaria Garozzo, Pasqua Betta, Salvatore Cilauro, Alessandro Saporito, Pietro D'Amico, Gabriella Tina, Angela Motta, Alfredo Pulvirenti, Salvatore Alaimo, Laura Sciuto, Basilio Pecorino, Manuela Ceccarelli, Guido Scalia, Tiziana Timpanaro, Martino Ruggieri, Agata Polizzi, Andrea D. Praticò

**Affiliations:** ^1^ Unit of Pediatrics and Pediatric Emergency, Cannizzaro Hospital, Catania, Italy; ^2^ Unit of Pediatric Clinic, Department of Clinical and Experimental Medicine, University of Catania, Catania, Italy; ^3^ Unit of Neonatal Intensive Care, University Hospital “Policlinico”, University of Catania, Catania, Italy; ^4^ Unit of Neonatal Intensive Care, Cannizzaro Hospital, Catania, Italy; ^5^ Unit of Neonatal Intensive Care, Garibaldi Hospital, Catania, Italy; ^6^ Bioinformatics Unit, Department of Clinical and Experimental Medicine, University of Catania, Catania, Italy; ^7^ Unit of Pediatrics, Lentini Hospital, Lentini, Italy; ^8^ Unit of Gynecology, Department of Medicine and Surgery, University Kore of Enna, Enna, Italy; ^9^ Unit of Infectious Diseases, Department of Medicine and Surgery, University Kore of Enna, Enna, Italy; ^10^ Laboratory Analysis Unit, Department of Biomedical and Biotechnological Sciences, University of Catania, Catania, Italy; ^11^ Unit of Pediatrics, Department of Medicine and Surgery, University Kore of Enna, Enna, Italy

**Keywords:** congenital toxoplasmosis, mother-to-child transmission, gestational age, neurological sequelae, prenatal screening

## Abstract

**Introduction:**

*Toxoplasma gondii* (*T. gondii*) primary infection during pregnancy can lead to severe consequences in the fetus and newborn, including miscarriage, congenital disease, or neuro-ophthalmological complications.

**Objectives:**

This study aimed to evaluate the incidence of congenital toxoplasmosis (CT) in a cohort of newborns and assess their neurological, ophthalmological, and auditory sequelae. Additionally, we examined correlations between infection rates, gestational age at maternal seroconversion, prenatal treatment, and postnatal outcomes.

**Methods:**

We studied a cohort of 220 newborns evaluated for suspected CT between 2000 and 2021 across three hospitals in Catania, Italy. Prenatal screening identified 98.6% of maternal infections. Collected data included gestational history, neonatal clinical data, and follow-up assessments.

**Results:**

Mother-to-child transmission (MTCT) occurred in 19.2% (29/151) of cases with available follow-up data. MTCT rates increased significantly with gestational age at maternal seroconversion: 5% in the first trimester, 23% in the second, and 63% in the third (*p* < 0.001). Prenatal treatment administered for ≥28 days was associated with a significantly lower MTCT rate (11.8% vs. 28.6%, *p* = 0.037). No significant association was found between maternal age and the risk of transmission (OR = 1.38, 95% CI: 0.54–3.55; *p* = 0.635). Of the 29 infected newborns, 17 (58.6%) were symptomatic at birth and during the long-term follow-up. Manifestations included microcephaly (10%), intracranial abnormalities (19%), behavioral disturbances (4%), epilepsy (7%), and psychomotor delay (7%). Ophthalmological lesions were present in 21% at birth and 45% during follow-up; no cases of hearing loss were recorded. No significant correlation was observed between gestational age at seroconversion and the presence of clinical symptoms, ocular findings, or neurological sequelae.

**Conclusions:**

Prenatal screening is effective in identifying newborns at risk for CT who require close monitoring and treatment. While our findings align with literature regarding MTCT rates, they differ regarding symptomatic case correlations. Further studies are warranted to better understand the factors influencing disease progression and long-term outcomes.

## Introduction

*Toxoplasma gondii (T. gondii)* is a globally distributed, obligate intracellular protozoan parasite that can infect humans and almost all warm-blooded animals. Members of the *Felidae* family serve as the definitive hosts (reservoir of the pathogen) and humans are intermediate hosts, where asexual reproduction occurs (1).

If acquired during pregnancy, primary infection can have devastating consequences. Mothers typically acquire the infection by ingesting oocysts from contaminated soil or water, or tissue cysts from undercooked infected meat. They are usually asymptomatic or present only mild, nonspecific symptoms, which are frequently unrecognized or unreported. As such, the infection is reliably detected only through seroconversion (1, 2). That is the reason why in many European Countries, including Italy, women are routinely screened during pregnancy with monthly search for anti-*Toxoplasma* antibodies if seronegative at the beginning, and receive an antepartum treatment once primary infection is diagnosed. According to a recent observational study in the province of Trento, Italy, the mean overall prevalence of *T. gondii* infection during pregnancy was 21.7%, with higher seroconversion rate among foreign women than Italian women (3). When antepartum treatment is promptly initiated (within 3 weeks after maternal seroconversion) both MTCT and risk of symptomatic congenital toxoplasmosis (CT) significantly decrease. According to the Italian consensus on management of CT, children born from mothers who seroconverted during pregnancy, have to follow a strict follow-up program, composed by laboratory, clinical, ophthalmological, auditive and imaging (brain ultrasound) examinations. In case of infection, the newborn-infant should start a prompt antiparasitic treatment, in order to prevent or reduce clinical signs of the disease. In the other cases, the condition of “not infected” should be established by the negativization of the IgG within the first year of age, confirmed twice (4, 5).

This study aims to evaluate the incidence of CT in a cohort of newborns and assess their neurological, ophthalmological, and auditory sequelae. Additionally, we examined correlations between infection rates, gestational age at maternal seroconversion, prenatal treatment, and postnatal outcomes.

## Subjects, materials and methods

We studied a cohort of 220 children born from mothers affected by primary *T. gondii* infection during pregnancy, retrospectively selected from 2000 to 2021 (Table 1). The study involved three third-level hospitals in the area of Catania: the “Policlinico-Vittorio Emanuele University Hospital”, with Neonatal Intensive Care Unit (*N*ICU) and Division of Pediatric Infectious Diseases, the “Cannizzaro Hospital”, with NICU, and the “Garibaldi Hospital”, with NICU. The data collection was executed in collaboration with the Department of Biomedical Science and Biotechnology of the University Hospital of Catania.

**Table 1:** Essay of each hospital and department to the data collection.

Department (Hospital)	Patients enrolled (n)
Division of Pediatric Infectious Diseases (Policlinico-Vittorio Emanuele University Hospital)	134
NICU (Garibaldi Hospital)	40
NICU (Cannizzaro Hospital)	23
NICU (Policlinico-Vittorio Emanuele University Hospital)	10
Department of Biomedical Science and Biotechnology (Policlinico-Vittorio Emanuele University Hospital)	13

The analyzed parameters were: gestational history, neonatal anamnesis and short and long-term follow-up. For women screened prenatally, we imputed seroconversion at the time of first positive immunoglobulin M (IgM) assay. Infected women were treated with spiramycin followed by pyrimethamine-sulfonamide or with spiramycin or pyrimethamine-sulfonamide alone, according to the trimester of seroconversion and to the positivity of the amniotic fluid *Toxoplasma* PCR. Prenatal treatment was defined as administration of anti-*Toxoplasma* therapy for a minimum of 28 consecutive days before delivery. This criterion was used to categorize neonates as “prenatally treated” or “non-prenatally treated” for the purpose of evaluating treatment impact on vertical transmission. Cases of reinfection, reactivation during pregnancy, abortion, and miscarriage were excluded.

Information on clinical, instrumental and laboratory findings were collected at birth, at first, second, third, sixth, ninth months and at first year of age, as suggested by Italian Multidisciplinary Team on Infectious diseases in Obstetric and Neonatology (3).

The laboratory examinations included the measurement of specific IgM and IgG levels using standard procedures throughout the follow-up period, the detection of IgM using the Immunosorbent Agglutination Assay (ISAGA), and the assessment of specific IgM and IgG by western blot analysis. The dosage of immunoglobulin by standard method was performed by the laboratory of each hospital, meanwhile the samples for ISAGA and western blot were centralized to the Department of Biomedical Science and Biotechnology of University Hospital of Catania. For the standard dosage of IgM and IgG the semiautomated method was performed (VIDAS® Toxo IgG II e Toxo IgM, bioMérieux® sa, Marcy l'Etoile, France). The cut-off values for seropositivity were 0.55 IU/ml and 35 IU/ml for IgM and IgG, respectively. For the dosage of IgM by ISAGA (Toxo-ISAGA IgM e Toxo-ISAGA IgA, bioMérieux® sa, Marcy l'Etoile, France) using a 1:20 dilution instead of 1:100. The western blot was performed by LDBIO diagnostics kit (Lyon, France), comparing mother's immunological response to child's one, at birth, and comparing child's present response with previous one, at first and second month of life. We established a database containing results of auxological and neurological assay, cerebral ultrasound, Computed Tomography (CT) Scan or Magnetic Resonance Imaging (MRI) ophthalmoscopic and audiometric evaluation, serological follow-up (anti *T. gondii* IgM and IgG titers) and information about postnatal treatment.

Diagnosis of CT was established based on one or more of the following criteria: (a) detection of *T. gondii* DNA by PCR in at least one fetal or neonatal compartment (e.g., amniotic fluid, CSF, blood, or urine); (b) persistent specific anti-*T. gondii* IgG titers beyond 12 months of age; (c) rising *T. gondii*-specific IgG titers during the first months of life; (d) presence of specific IgM and/or IgA in neonatal serum, confirmed by ISAGA or Western blot; or (e) compatible clinical findings (neurological, ophthalmological, systemic) in association with positive serology.

Cases not fulfilling these criteria and lost to follow-up were classified as “unknown.”

## Statistical analyses

All statistical analyses were conducted using SPSS Statistics version XX (IBM Corp., Armonk, NY). A two-sided *p*-value <0.05 was considered statistically significant.

Continuous variables were described as means ± standard deviation (SD) or medians with interquartile ranges (IQR), depending on their distribution. Categorical variables were summarized using counts and percentages. The Wilcoxon rank-sum test was used to compare birth outcomes and serological markers (e.g., IgG, IgM) between treatment groups and across trimesters of maternal seroconversion. The Chi-square test (or Fisher's exact test when appropriate) was applied to assess associations between categorical variables, including maternal treatment type and MTCT rate.

Univariate logistic regression was used to estimate odds ratios (ORs) with 95% confidence intervals (CIs) for predictors of MTCT and clinical manifestations at birth or during follow-up.

Separate multivariate logistic regression models were then constructed for each of these three outcomes. The following independent variables were included in all models: (a) Gestational age at maternal seroconversion (first, second, or third trimester); (b) Prenatal treatment type (spiramycin alone, P/S alone, or sequential combination); (c) Maternal age (<29 vs. ≥29 years). Only patients with complete outcome data were included in each respective model. Due to heterogeneity in follow-up length and incomplete documentation in some cases, follow-up duration was not used as a covariate in the analysis of long-term outcomes.

## Results

### Study population and follow-up

Of the 220 enrolled pregnancies, 69 cases (31.4%) had insufficient follow-up data to determine the infection status of the newborn, primarily due to loss to follow-up. These cases were excluded from all outcome-related analyses. Among the remaining 151 mother–infant pairs with known infection status, the overall rate of mother-to-child transmission (MTCT) was 19.2% (29/151), with 58.6% (17/29) of infected infants presenting with symptoms at birth.

### Maternal seroconversion, treatment and comorbidities

**Figure 1:**
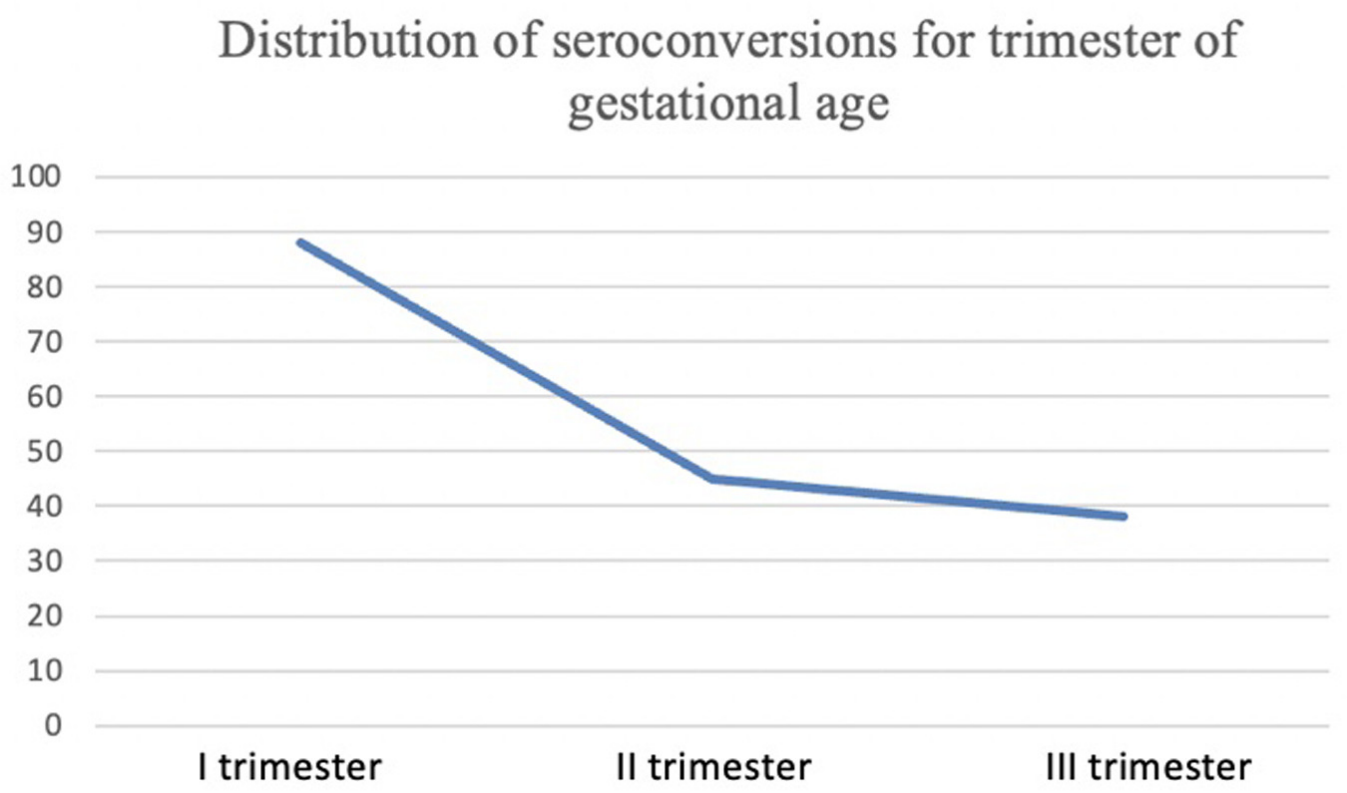
Number of women identified by prenatal screening who seroconverted during first, second and third trimester of gestational age.

The largest part (40%) of women identified by prenatal screening seroconverted during first trimester of gestational age (Figure 1); 95.3% of women received treatment, with a predilection for spiramycin in monotherapy. Despite the lack of data, 10.9% of women was estimated not to have strictly followed the treatment prescription of obstetrician and/or microbiologist. Only five women underwent amniocentesis, with no detection of parasite in the amniotic fluid; therefore, because the lack of data we were not able to correlate the amniotic fluid parasitic load with MTCT. The most common comorbidities of pregnant women were arterial hypertension, oligohydramnios and diabetes; none was immunosuppressed (Table 2).

**Table 2:** Characteristic of gestational age in study population.

Characteristic	Prenatal screened women (*n*)			
	I trimester	II trimester	III trimester	Unknown
Gestational age at maternal seronconversion	88	45	38	49
Age	<20 years	20–29 years	>29 years	Unknown
	10	34	33	143
Prenatal treatment	Yes	No		Unknown
	178	28		14
Type of treatment	Spiramycin	P/S	Spiramycin + P/S	Unknown
	67	4	61	88
Compliance to treatment	Good	Bad		Unknown
	1	24		195
Performance of amniocentesis	Yes	No		Unknown
	5			205
PCR for *T. gondii* in amniotic fluid	Positive	Negative		
		5		
Comorbidities	Positive			
	13			

P/S, Pyrimethamine-Sulphonamide.

Maternal comorbidity: intrauterine fetal death (*n* = 1), HBV infection (*n* = 1), Parvovirus B19 infection (*n* = 1), hypertransaminasemia (*n* = 1), oligohydramnios (*n* = 2), diabetes (*n* = 2), arterial hypertension (*n* = 2), Antithrombin III deficiency (*n* = 1), gestosis (*n* = 1).

When stratifying the population of women who seroconverted during pregnancy by age, 12.2% were younger than 20 years, 56.8% were between 20 and 29 years, and 31.0% were 30 years or older. The highest proportion of seroconversions occurred in the 20–29-year age group. No significant association was observed between maternal age and the risk of mother-to-child transmission (OR = 1.38 for ≤29 years vs. >29 years; 95% CI: 0.54–3.55; *p* = 0.635).

The rate of MTCT increased with later seroconversion. The estimated rate of MTCT by gestational age was 5% at first trimester (OR = 0.12, 95% CI: 0.03–0.45), 23% at the second (OR = 1.18, 95% CI: 0.42–3.28) and 63% at the third trimester (OR = 5.80, 95% CI: 1,99–16-87), with a linear increase of infection rate for every week of gestational age (Table 3) and a *p*-trend <0.01. In multivariate analysis, gestational age remained a significant independent predictor of MTCT (OR = 1.38 for ≤29 years vs. >29 years; 95% CI: 0.54–3.55; *p* = 0.635) (Table 3). When reanalyzed based on the defined criterion for prenatal treatment (i.e., at least 28 consecutive days of therapy before delivery), the MTCT rate was significantly lower in the treated group (15.2%, 21/138) compared to those untreated or treated for <28 days (34.8%, 8/23) (OR = 0.34, 95% CI: 0.13–0.89, *p* = 0.037). The type of treatment (spiramycin vs. P/S vs. combination) did not significantly affect MTCT (OR for combination vs. monotherapy 1.88, 95% CI: 0.64–5.56, *p* = 0.254).

**Table 3:** Contingency analysis of mother-to-child transmission (MTCT) by trimester of maternal seroconversion, maternal Age, and prenatal treatment. Patients with insufficient data were excluded.

Variable		MTCT	No MTCT	Total	*p*
Trimester of gestation	1st	2	86	88	<0.01
	2nd	9	36	45	
	3rd	18	20	38	
Maternal age (years)	≤29	18	26	44	0.635
	>29	11	22	33	
Prenatal Treatment >28 days	Yes	21	117	138	0.037
	No	8	15	23	

### Prenatal brain ultrasound findings

Regarding pre-natal brain ultrasound, results were available in 125 patients out of 220 (56.8%). Among these, 34 (27.2%) had positive results (i.e., periarteritis, periventricular hyperechogenicity, intraventricular hemorrhage, asymmetry of lateral ventricles, bilateral ventriculomegaly, frontal cyst, hyperechogenicity of the caudate nucleus, germinal zone hemorrhage, intraventricular hemorrhage). Among the 29 infected, data were available in 21 (72.4%), and a positive brain ultrasound finding was found in 13 (61.9%). The occurrence of brain ultrasound findings was statistically significantly more common in infected patients (*p* = 0.002).

### Neonatal clinical and serological characteristics

**Figure 2:**
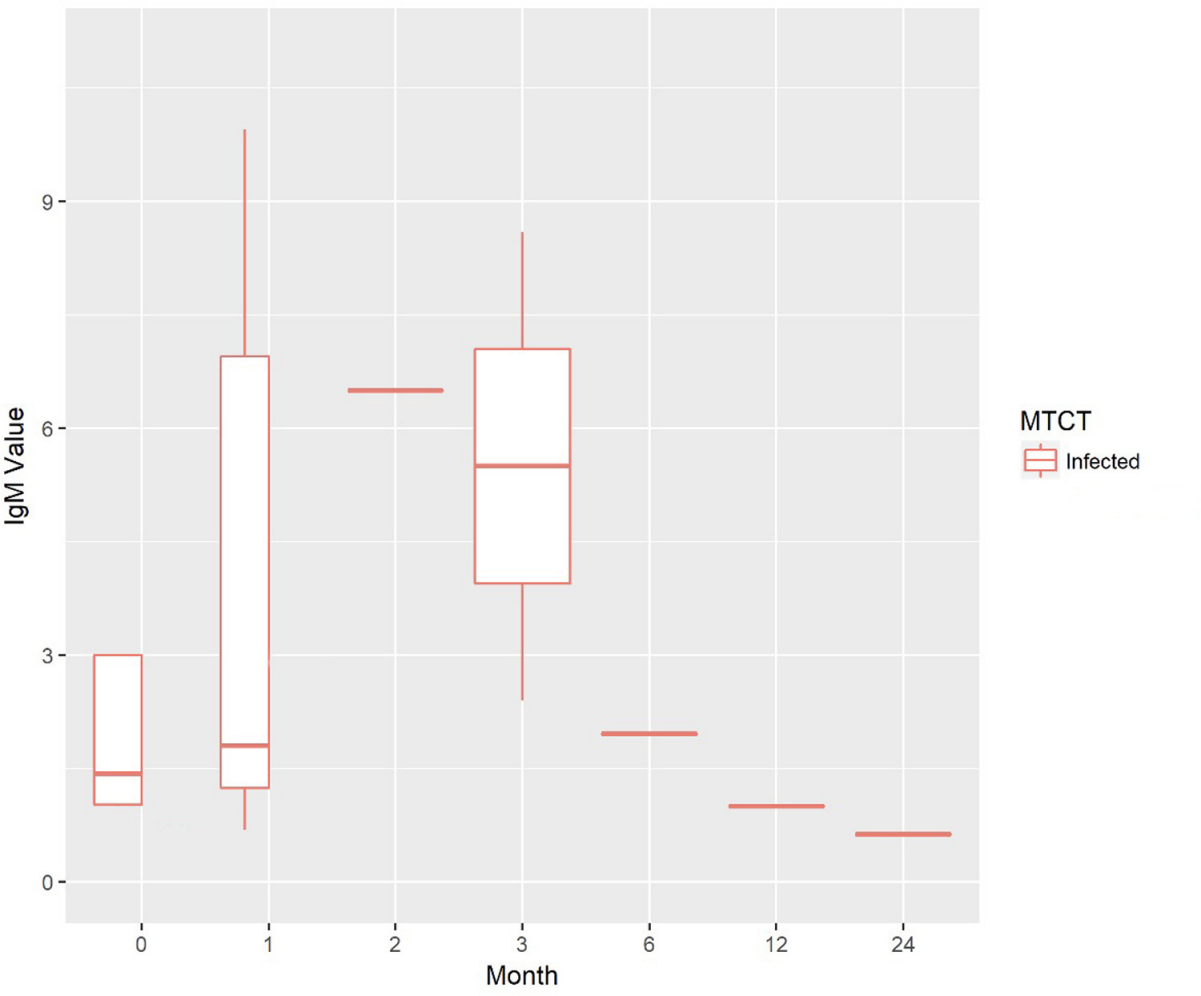
Trend of IgM values during the newborns/infants' follow-up.

**Figure 3:**
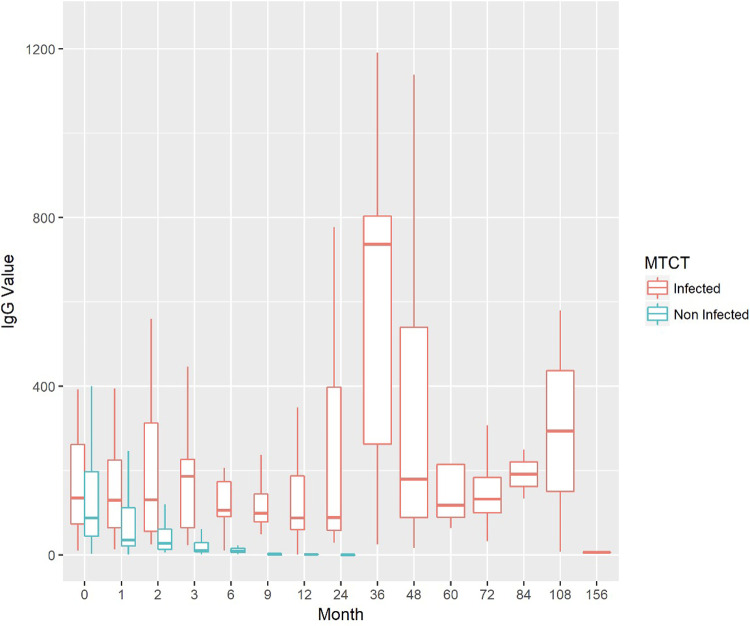
Trend of IgG values during the newborns/infants' follow-up.

Of the newborns, 52.7% were male. Mean birth weight was 3,072 g. IgM seropositivity was found in 37.9% of infected neonates. In 43.3% of cases (74/171) the dosage of immunoglobulins was performed from funicular artery blood, in 56.7% (97/171) from venous blood; in the remaining cases, the origin of blood samples was unknown. The average value of IgG at birth was 195.9 IU/ml (SDs 467.6; median value 78 IU/ml). Figures 2, 3 show the trend of IgM and IgG respectively in all the cohort of patients enrolled, during the entire follow-up. Western blot (WB) analysis was performed in 106 neonates to support diagnostic clarification, particularly in cases with discordant or inconclusive IgM results. Among the 29 infected children, WB was available in 26 cases (89.7%) and identified 25 cases (sensitivity 96.15%).

**Figure 4:**
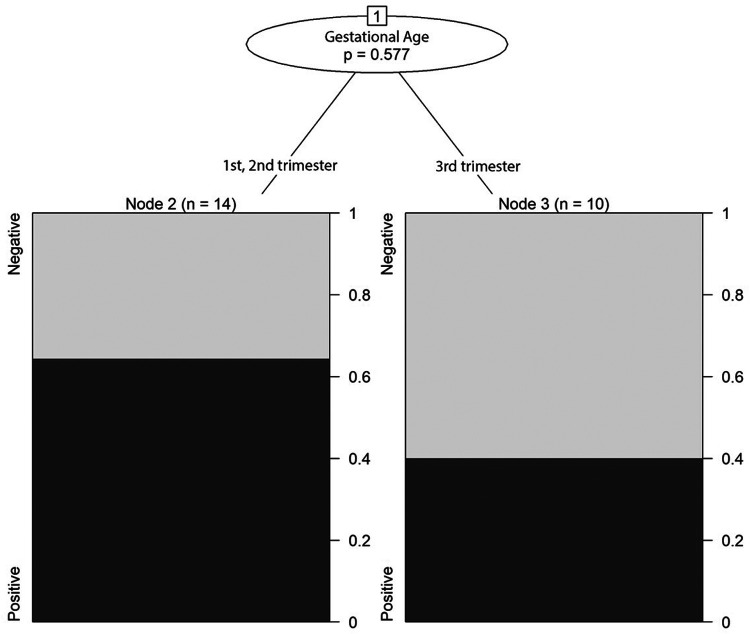
Correlation between trimester of gestational age at seroconversion and prevalence of ocular manifestations.

The largest part of newborns selected was born at term, without asphyxia, with normal weight (average percentile 43.3) and normal head circumference (average percentile 41.8). Among infected newborns, 3.4% showed systemic signs (e.g., hypertransaminasemia, anaemia), 24.1% ocular or brain involvement at birth (Table 4). A later gestational age at seroconversion was not associated with higher odds of symptomatic infection (OR = 0.93, 95% CI: 0.41–2.10, *p* = 0.93) or neurological involvement (OR = 0.91, 95% CI: 0.18–4.67, *p* = 0.913). A trend toward reduced ocular involvement with later seroconversion was noted, though not statistically significant (OR = 0.20, 95% CI: 0.01–2.87, *p* = 0.577) (Figure 4).

**Table 4:** Main demographic characteristics of the patients.

Characteristic	Newborn/children (*n*)		
Gender	Male	Female	
	116	104	
Gestational age at birth	Premature	Born at term	Unknown
	21	166	33
Route of delivery	Natural childbirth	Cesarean childbirth	Unknown
	78	81	51
Weight at birth	Normal	Underweight	Unknown
	177	9	34
Brain circumference at birth	Normal	Microcephaly	Unknown
	12	4	204
APGAR score at first minute	Normal	Depressed	Seriously depressed
	106	6	

### Clinical presentation and long-term outcomes in infected infants

Focusing solely on the 29 infected children, we compared those who received ≥28 days of prenatal treatment (*n* = 21) to those untreated or treated <28 days (*n* = 8). At birth, symptomatic infection was observed in 52.4% of treated vs. 75.0% of untreated children (*p* = 0.40), while neurological manifestations were present in 19.0% vs. 37.5%, and ocular involvement in 23.8% vs. 37.5%, respectively. During follow-up, at least one complication (ocular or neurological) was observed in 47.6% of treated vs. 62.5% of untreated cases (*p* = 0.47).

Long-term follow-up data were available for 25 of the 29 infected children, with a median follow-up duration of 3.4 years (IQR: 2.1–5.0 years). During this period, 13/29 (44.8%) developed chorioretinitis; with 8 (27.6%) progressing to severe impairment (e.g., contralateral chorioretinitis, strabismus, visual impairment). Among the neurological manifestations, 3 manifested microcephaly, 5 intracranial lesions (i.e., periventricular leukomalacia, or calcifications) detected by postnatal cranial ultrasound, 3 confirmed on brain MRI (i.e., white matter abnormalities or calcifications). Moreover, only 2 developed epilepsy, and 3 psychomotor delay and behavioral disturbances. No cases of hearing loss were observed. These results were not associated with the timing of treatment initiation (Table 5), with the exception of severe ocular impairment after the first year of life, which showed a correlation with an earlier treatment.

**Table 5:** Association between clinical outcomes and timing of prenatal treatment initiation in patients with congenital toxoplasmosis.

Variable	Present		Absent		*p*
	Patients	Average time after treatment initiation in weeks (SDS)	Patients	Average time after treatment initiation in weeks (SDS)	
Chorioretinitis in the first 6 months of life	13/29	1.4 (0.32)	16/29	1.2 (0.39)	NS
Severe ocular improvement after 1 year of life	8/29	1.65 (0.35)	21/29	1.13 (0.32)	0.01
Microcephaly	3/29	1.68 (0.32)	26/29	1.33 (0.36)	NS
Intracranial lesions (periventricular leukomalacia/calcifications)	5/29	1.4 (0.33)	24/29	1.34 (0.31)	NS
Epilepsy	2/29	1.54 (0.36)	27/29	1.36 (0.32)	NS
Psychomotor delay/behavioral disturbances	3/29	1.56 (0.31)	26/29	1.32 (0.29)	NS

The majority of infected children was treated with P/S continued until the first year of life. Adverse effects related to postnatal treatment were observed in two patients: one developed seizures and another nephrolithiasis, both requiring treatment discontinuation.

No systematic data on prenatal treatment tolerability were available. Cases of reactivation of retinochoroiditis were treated with the association of trimethoprim and sulfametoxazol, which has been shown to reduce the risk of recurrence in individuals of ocular toxoplasmosis.

## Discussion

This retrospective cohort study provides updated data on the incidence and clinical spectrum of congenital toxoplasmosis in a large Sicilian population. We confirmed the strong influence of gestational age at maternal seroconversion on the risk of vertical transmission, with significantly higher rates in third-trimester infections. Timely and adequate prenatal treatment was associated with a reduced MTCT rate, though not with lower rates of long-term complications. Prenatal brain ultrasound abnormalities were significantly more frequent in infected neonates.

In our cohort of 151 patients with sufficient pregnancy data, we observed a MTCT rate of 19.2%, which is lower than the 29.4% reported by the SYROCOT study group in 2007 (6). This difference may be partially explained by increased awareness and improved prenatal screening and treatment practices in the more recent period covered by our study. Notably, an unexpectedly low number of amniocenteses (5/220) were performed in our cohort, despite the procedure being recommended by most clinical guidelines following confirmed maternal infection (2, 4, 5). This underuse may reflect several factors, including perceived procedural risks, patient or clinician hesitancy, inconsistent application of protocols across centers, and evolving clinical practices over the 20-year study period.

Maternal age ≥29 years was not significantly associated with MTCT risk (OR = 1.38; 95% CI: 0.54–3.55; *p* = 0.635), though fewer seroconversions in older women are consistent with the known increase in seroprevalence with age (1). In our cohort, most seroconversions occurred in the 20–29-year group, supporting the hypothesis that younger women, with lower baseline immunity, are more susceptible to primary infection during pregnancy. However, since our study included only women who seroconverted during pregnancy, we could not directly assess age-related seroprevalence in the general obstetric population. A population-based design would be required to explore this relationship more accurately (1, 3). The SYROCOT study (6) also showed that the risk of clinical manifestations, especially intracranial lesions, decreases with advancing gestational age at seroconversion, whereas the decline for ocular lesions was less pronounced.

Our multivariate analysis confirmed that gestational age at maternal seroconversion is a major predictor of MTCT. Specifically, MTCT risk increased progressively across trimesters: 5% in the first, 23% in the second, and 63% in the third trimester (OR for third vs. first trimester = 6.72; 95% CI: 2.07–21.78; *p* = 0.002). These findings support previous literature demonstrating a higher probability of vertical transmission with advancing gestational age, regardless of prenatal treatment (1, 6).

Regarding the effect of specific prenatal treatment strategies on MTCT, when differentiating among spiramycin monotherapy, pyrimethamine-sulphonamide (P/S) monotherapy, and sequential spiramycin followed by P/S, no statistically significant differences were found in either univariate or multivariate models (adjusted OR for sequential treatment vs. monotherapy = 1.88; 95% CI: 0.64–5.56; *p* = 0.254). While these findings may appear in line with those of the SYROCOT study (6), it is important to acknowledge the methodological limitations of that meta-analysis. More importantly, the lack of statistical significance in our study is likely due to limited sample size and reduced statistical power. A potentially greater efficacy of pyrimethamine-sulfonamide compared to spiramycin was already suggested in the TOXOGEST study (7) and has recently been reinforced by a systematic review and meta-analysis by Ribeiro et al., which confirmed that prenatal treatment significantly reduces both vertical transmission rates and the risk of clinical manifestations at birth, particularly when initiated early and maintained for a sufficient duration. Their findings provide additional evidence supporting prenatal therapy as a central component of CT prevention strategies and further validate the clinical relevance of our results (8), that—though limited by sample size—similarly indicate that treatment duration may be more influential than regimen type in modulating the risk of MTCT and long-term outcomes. In our cohort, when applying the threshold of ≥28 consecutive days of treatment, MTCT was significantly reduced in the treated group (*p* = 0.037), supporting the benefit of adequate treatment duration.

In addition to transmission rates, we also evaluated the association between prenatal treatment and disease severity among infected infants. Although statistical significance was not reached, our findings suggest a trend toward lower rates of clinical manifestations at birth and during follow-up in those receiving ≥28 days of prenatal therapy. This observation aligns with prior evidence that prenatal treatment may attenuate disease severity in addition to reducing transmission risk, as recently demonstrated in cohort studies from Spain and Brazil (9, 10), which reported a significant association between prenatal therapy and reduced symptomatology at birth. The lack of statistical significance in some of our subgroup analyses may reflect limited statistical power rather than a true absence of effect.

We found, as expected, a progressive reduction of specific serological IgG in infant treated for CT, followed by a rebound of antibody titer after the interruption of therapy. According to the literature, typical patterns of serologic evolution reflect the combined effects of elimination of passively acquired maternal IgG and active production of IgG by the infant. Reductions in IgG titers, in some instances transiently declining to undetectable, are observed during treatment, followed by a rebound when treatment is discontinued (11–16). According to some authors the development of ocular lesions may be not associated with a change in antibody titers (1). On the contrary we observed that some serological rebound forewarns ophthalmologic lesions in infected patients.

In non-infected infants, IgG negativity was achieved by the ninth month of life in the majority of cases. This finding is consistent with existing literature, which consistently supports that the criterion for excluding CT is the complete and sustained loss of specific IgG antibodies within the first year of life (1).

Regarding the IgM antibody assay, 37.9% of infected newborns were seropositive at birth, while no cases of IgM seropositivity were detected beyond the third month of life. The diagnostic performance of IgM detection in neonatal CT remains controversial, with reported sensitivity ranging from 54% to 76.5% (17, 18), and likely influenced by factors such as gestational age at maternal seroconversion and the presence of prenatal treatment. Prenatal treatment is known to reduce parasitemia and antigen load, which can in turn decrease the sensitivity of both IgM detection and PCR at birth. This may lead to false-negative results, especially in infants who received adequate intrauterine therapy. Consequently, a negative IgM result at birth does not definitively exclude congenital infection, particularly in treated neonates. This underscores the importance of a structured follow-up program, including repeated serologic testing and clinical evaluations over time, to reliably confirm or rule out disease.

Some Authors recommend performing the test on peripheral blood within the first two weeks of life, as its sensitivity may be affected by maternal gestational age at seroconversion and by prenatal treatment (19, 20). In our cohort, we were unable to correlate IgM seropositivity with maternal factors due to limited available data.

The western blot (WB) assay demonstrated a more reliable performance in our study, with only one false-negative result, demonstrating a satisfactory sensitivity (96.15%). The WB method is considered a valuable complement to conventional diagnostic tools in the early detection of CT, with a reported sensitivity of up to 82.6% (21).

Our data also indicate that the majority (80%) of infected children were asymptomatic at birth, but symptoms developed in 59% during long-term follow-up, including neurological and ophthalmologic complications. This reinforces the importance of ongoing monitoring, even in asymptomatic neonates. Our study concurs with a large study by Di Carlo et al., who found a 57% prevalence of features of *T. gondii* infection in a follow-up of at least 2 years (median 4.6 years; range 2–6 years) (22). No correlation between congenital infection and prematurity or intrauterine growth retardation was observed in the present study. The association of CT with prematurity and intrauterine growth retardation remains controversial (22–29).

Among the neurological signs observed, 10% had microcephaly, 19% had brain abnormalities on ultrasound at birth, 4% showed behavioral disturbances, 7% developed epilepsy, and 7% had psychomotor delay with behavioral issues (Table 6). Compared to the lower rates of serious neurological sequelae reported by Cortina-Borja et al. (30), this difference may reflect broader definitions used in our study. Their criteria included microcephaly, shunt placement, seizures, neurodevelopmental abnormalities requiring referral, severe bilateral visual impairment, cerebral palsy, or death before 2 years of age. Unlike the SYROCOT meta-analysis (6), which found an inverse correlation between gestational age at maternal seroconversion and intracranial lesions, our multivariate analysis did not show any significant association between trimester of infection and neurological, ocular, or overall clinical manifestations. These discrepancies may be due to limited statistical power and our smaller sample size. Our findings suggest that gestational timing may not be a strong predictor of disease severity, although larger studies are needed to confirm this.

**Table 6:** Multivariate logistic regression analyses for main outcomes.

Outcome	Predictor	Adjusted OR (95% CI)	*p*-value
Mother-to-child transmission	2nd vs 1st trimester seroconversion	1.11 (0.38–3.27)	0.844
	3rd vs 1st trimester seroconversion	6.72 (2.07–21.78)	0.002
	Combined vs monotherapy treatment	1.88 (0.64–5.56)	0.254
	Maternal age ≥29 years	0.33 (0.08–1.39)	0.124
Symptomatic at birth	3rd vs 1st trimester seroconversion	0.93 (0.41–2.10)	0.930
	Combined vs monotherapy treatment	0.78 (0.27–2.25)	0.645
Ocular manifestations	3rd vs 1st trimester seroconversion	0.20 (0.01–2.87)	0.577
Neurological involvement	3rd vs 1st trimester seroconversion	0.91 (0.18–4.67)	0.913

We observed a rate of ophthalmological lesions higher than in literature: 21% vs. 12% (9) and 6.4% (31) at birth and 45% vs. 17% (32) and 29% (31) in the long-term follow-up. The significant difference between our rate of ophthalmological lesions and what reported in literature in the long-term follow-up can be explained by the different type of study: retrospective vs. prospective. Then, also in this case, definitions are different: Tan et al. studied subjects with congenital *T. gondii* retinochoroiditis (32), while we reported also the minor ophthalmological lesions, as microphthalmia, strabismus and cataract.

No cases of hearing loss were found. Given that congenital infection can be considered as an important risk factor for hearing impairment, the data about the efficacy of postnatal treatment in improving/solving sensorineural hearing loss are contradictory. Andrade et al. observed the persistence of hearing impairment in half of congenitally infected and treated children (33); conversely, McGee et al. described no infant or child with sensorineural hearing loss among treated ones (34) and Salomè et al. observed only one patient with sensorineural hearing loss at the onset but apparently unrelated to CT and no patients with any grade of delayed hearing loss both in treated and untreated group (35).

The standard postnatal treatment is pyrimethamine combined with sulfonamide, with each country adopting its own protocols for dosage and duration; in Italy, a 12-month regimen is recommended. Spiramycin, once used in neonates, is no longer advised due to its association with arrhythmias. In our cohort, trimethoprim/sulfamethoxazole was administered in selected cases of reactivated retinochoroiditis during long-term follow-up. While not a first-line treatment, this combination is considered a viable alternative for secondary prophylaxis due to its oral route, tolerability, and lower toxicity, and has been associated with reduced recurrence risk (36–38).

## Study limitations

This study has several limitations that should be acknowledged. First, due to its retrospective design, certain key clinical and laboratory data were not consistently available across all cases, limiting the completeness of some analyses. In particular, we were unable to determine the precise timing of prenatal treatment initiation, making it impossible to assess whether therapy was administered within the recommended “window of opportunity” (i.e., within 28 days of maternal diagnosis). Second, prenatal imaging data, including fetal ultrasound findings, were not systematically recorded and therefore could not be analyzed. Third, the small number of women undergoing amniocentesis prevented us from evaluating the association between amniotic fluid parasitic load and risk of vertical transmission. Fourth, although we classified treatment types and regimens, the actual duration and compliance with prescribed therapies could not be reliably assessed in all patients; additionally, side effects related to prenatal therapy were not systematically recorded in clinical documentation, limiting our ability to evaluate the safety profile of *in utero* treatment. Data on postnatal treatment-related adverse events were limited to cases with explicitly documented complications. Fifth, the lack of IgA serology testing at birth limited our ability to evaluate its diagnostic utility in conjunction with IgM and IgG. Sixth, while the study explored the association between prenatal treatment and the presence of clinical signs at birth, it was not designed to assess whether such treatment had a protective effect against the development of new complications during long-term follow-up. This important limitation should be considered when interpreting the clinical impact of prenatal therapy, as the lack of follow-up-based outcome evaluation may underestimate its true benefit. Seventh, the relatively small number of infected children may have limited the statistical power to detect significant differences between groups, particularly in subgroup analyses related to treatment efficacy.

Eighth, it was not possible to perform a multivariate analysis adjusted for the gestational age at seroconversion, a known independent predictor of transmission and clinical severity. This further limits the interpretability of observed associations. Additionally, follow-up duration varied across cases and was not uniformly documented, which may have influenced the detection of long-term sequelae. Ninth, we could not correlate parasite genotype with clinical severity, as molecular typing was not available. Finally, the study cohort was limited to a single region, which may affect the generalizability of findings to other populations with different screening policies, dietary habits, and circulating genotypes.

## Conclusions

Prenatal serological screening remains a cornerstone in identifying newborns at risk for congenital toxoplasmosis (CT), enabling early follow-up and timely treatment. Our findings confirm a strong association between gestational age at maternal seroconversion and the risk of mother-to-child transmission (MTCT), with significantly higher transmission rates in third-trimester infections. Importantly, we observed that prenatal treatment administered for at least 28 days significantly reduced the rate of MTCT, reinforcing the clinical value of both routine screening and prompt therapeutic intervention. Although no correlation was found between gestational age at seroconversion and disease severity, our findings support the protective role of prenatal therapy against vertical transmission. Despite the existence of a national consensus on CT management, significant heterogeneity persists across Italian regions, highlighting the need for more standardized implementation protocols and potentially for a national registry of congenital infections.

## Data Availability

The datasets presented in this study can be found in online repositories. The names of the repository/repositories and accession number(s) can be found in the article/Supplementary Material.
